# Phylogeographic analyses point to long-term survival on the spot in micro-endemic Lycian salamanders

**DOI:** 10.1371/journal.pone.0226326

**Published:** 2020-01-13

**Authors:** Michael Veith, Bayram Göçmen, Konstantinos Sotiropoulos, Karolos Eleftherakos, Stefan Lötters, Olaf Godmann, Mert Karış, Anil Oğuz, Sarah Ehl

**Affiliations:** 1 Department of Biogeography, Trier University, Universitätsring, Trier, Germany; 2 Ege University, Faculty of Science, Department of Biology, Zoology Section, Bornova, İzmir, Turkey; 3 Department of Biological Applications & Technology, University of Ioannina, Ioannina, Greece; 4 Section of Zoology-Marine Biology, Department of Biology, University of Athens, Athens, Greece; 5 Hauptstraße, Niedernhausen, Germany; Universita degli Studi della Tuscia, ITALY

## Abstract

Lycian salamanders (genus *Lyciasalamandra*) constitute an exceptional case of micro-endemism of an amphibian species on the Asian Minor mainland. These viviparous salamanders are confined to karstic limestone formations along the southern Anatolian coast and some islands. We here study the genetic differentiation within and among 118 populations of all seven *Lyciasalamandra* species across the entire genus’ distribution. Based on circa 900 base pairs of fragments of the mitochondrial 16SrDNA and ATPase genes, we analysed the spatial haplotype distribution as well as the genetic structure and demographic history of populations. We used 253 geo-referenced populations and CHELSA climate data to infer species distribution models which we projected on climatic conditions of the Last Glacial Maximum (LGM). Within all but one species, distinct phyloclades were identified, which only in parts matched current taxonomy. Most haplotypes (78%) were private to single populations. Sometimes population genetic parameters showed contradicting results, although in several cases they indicated recent population expansion of phyloclades. Climatic suitability of localities currently inhabited by salamanders was significantly lower during the LGM compared to recent climate. All data indicated a strong degree of isolation among *Lyciasalamandra* populations, even within phyloclades. Given the sometimes high degree of haplotype differentiation between adjacent populations, they must have survived periods of deteriorated climates during the Quaternary on the spot. However, the alternative explanation of male biased dispersal combined with a pronounced female philopatry can only be excluded if independent nuclear data confirm this result.

## Introduction

Small-range endemism at both species and higher taxonomic level is a common phenomenon among all three amphibian orders. Various mechanisms exist leading to small ranges and isolation (e.g., [1, 2]). We here put light on Lycian salamanders (*Lyciasalamandra*), an old clade of the Salamandridae, which has evolved high diversity of allopatric micro-endemic lineages [3].

Lycian salamanders occur in the western Taurus Mountains along the Mediterranean coast of Turkey and on the Greek Karpathos Archipelago. Seven species, some with various subspecies, have been suggested. Each is endemic to an isolated mountain ridge of its own, with almost no signs of admixture among each other even when they live in close proximity [4]. *Lyciasalamandra* is the sister genus of *Salamandra* and evolved circa 9.29 million years ago (mya) (95% confidence interval (CI): 6.12–12.8 mya; [5]). The final emergence of the mid-Aegean trench 10.2–12.3 mya probably initiated species evolution within *Lyciasalamandra* [6]. Phylogenetic studies consistently support a scenario of largely simultaneous emergence of seven major lineages [7, 8], and the hypothesis of a hard polytomy could in fact not be rejected [6].

Intraspecific evolution within *Lyciasalamandra* species can be explained by the Messinian Salinity Crisis (5.3–5.6 mya) as well as repeated climatic alterations since the Late Pliocene and throughout the Pleistocene [5, 6]. Given this high evolutionary age of *Lyciasalamandra* species and their intraspecific lineages [6] in combination with a low level of genetic diversity of local populations and a high amount of private local mitochondrial DNA haplotypes [8], it is sound to assume that these lineages, once having evolved, were not able to substantially disperse out of their mountains. Repeated bottlenecks associated with an increasing effect of genetic drift may have contributed to or even reinforced this pattern of low degrees of local genetic variability. And if eventually small-scale dispersal may have occurred, such founder events again should have reduced local genetic variability. All this may have led to today’s small-scale differences of pattern and colour among populations, forming the basis for the description of numerous new taxa within the last years (e.g., [9–16]). Unfortunately, up to know data supporting such a scenario solely stem from organelle data, so male-biased dispersal, which may erode local population differentiation, can only be detected by also analysing nuclear DNA.

But how could these salamanders survive such long periods ‘on the spot’, especially with regard to the tremendous climatic alterations which repeatedly occurred during the last 2.4 my [17]? Especially, since that time, cold periods (stitials) and warm (interstitials) periods regularly alternated [18]. This is known to have drastically affected the spatial distribution of plants and animals in the entire Western Palearctic (e.g., [19, 20]). Although ice ages along the Mediterranean coast may not have been as severe as in the interior land masses [21], it is suggested that the increasingly dry conditions during glacial periods had an impact on amphibian populations [22, 23].

The ecological niche of the enigmatic Lycian salamanders might be the key to understand this unique situation. Almost all known populations live on karstic limestone. Throughout the entire Mediterranean, such rock formations are associated with an increased biological diversity because they offer a variety of micro-habitats [24]. Especially at the slopes of south-facing coastal mountains karstic limestone provides a sufficient moisture gradient to organisms due to the opportunity to deeply hide inside crevices of rock formations and boulder fields. As major pre-requisite of this life-style, the Lycian salamanders had evolved a viviparous mode of reproduction, with females giving birth to only two juveniles per year.

Relying on stable micro-climatic conditions through vertical movements in order to constantly follow suitable micro-habitat conditions within short distances (e.g., [25]) may also have constrained Lycian salamanders to evolve a high degree of ecological plasticity. Rödder et al. [26] demonstrated that the climate niche of six *Lyciasalamandra* species is similar, with merely the single south-east Aegean Sea Island (i.e. non-coastal) species, *L*. *helverseni*, showing a deviating climate niche. Further support for intra-generic climate niche conservatism comes from a study on demographic life-history parameters. Sinsch and co-authors [27] found that detectable differences in life history traits among populations of different species and subspecies are mainly due to variation in the period of surface activity rather than being the result of fundamental differences in their ecological adaptation.

On the one hand, the highly specialised ecological and reproductive adaptations of *Lyciasalamandra* species allowed them to survive in primarily hostile environments. On the other hand, this may have ‘trapped’ them on suitable habitat spots, with restricted options to disperse and to cross areas lacking suitable micro-habitats. Such rare colonization events would result in low levels of within and high levels of among population genetic differentiation (e.g., [8]). Up to now, evidence for restricted gene flow among *Lyciasalamandra* populations comes from studies that were mainly conducted to solve their inter- and intra-specific phylogenetic relation (e.g., [6–8]). Accordingly, sample designs were not elaborated to infer evolutionary processes at smaller geographic scales.

We here apply a population sampling across all species to test the hypothesis that most *Lyciasalamandra* populations are strongly isolated from each other. In the absence of strong male-biased dispersal, this would be shown by the existence of numerous local haplotypes and by low levels of genetic (haplotype) diversity within and high levels of haplotype diversity among populations. This prediction is based on the assumption that the pronounced adaptation of Lycian salamanders to a largely subterranean life style, coupled with viviparity, has continuously trapped them inside once colonised mountains. By applying species distribution models (SDMs) and using current and past climate scenarios, we therefore test a second hypothesis that these salamanders do not need to horizontally move out of trouble in times of drastically changed climatic conditions (as reflected by the Last Glacial Maximum (LGM)), which we use as a proxy for the numerous climatic alterations that repeatedly occurred during the Pleistocene; rather were they able to survive on the spot during periods of cold and dry climate.

## Materials and methods

### Population sampling

**Fig 1:**
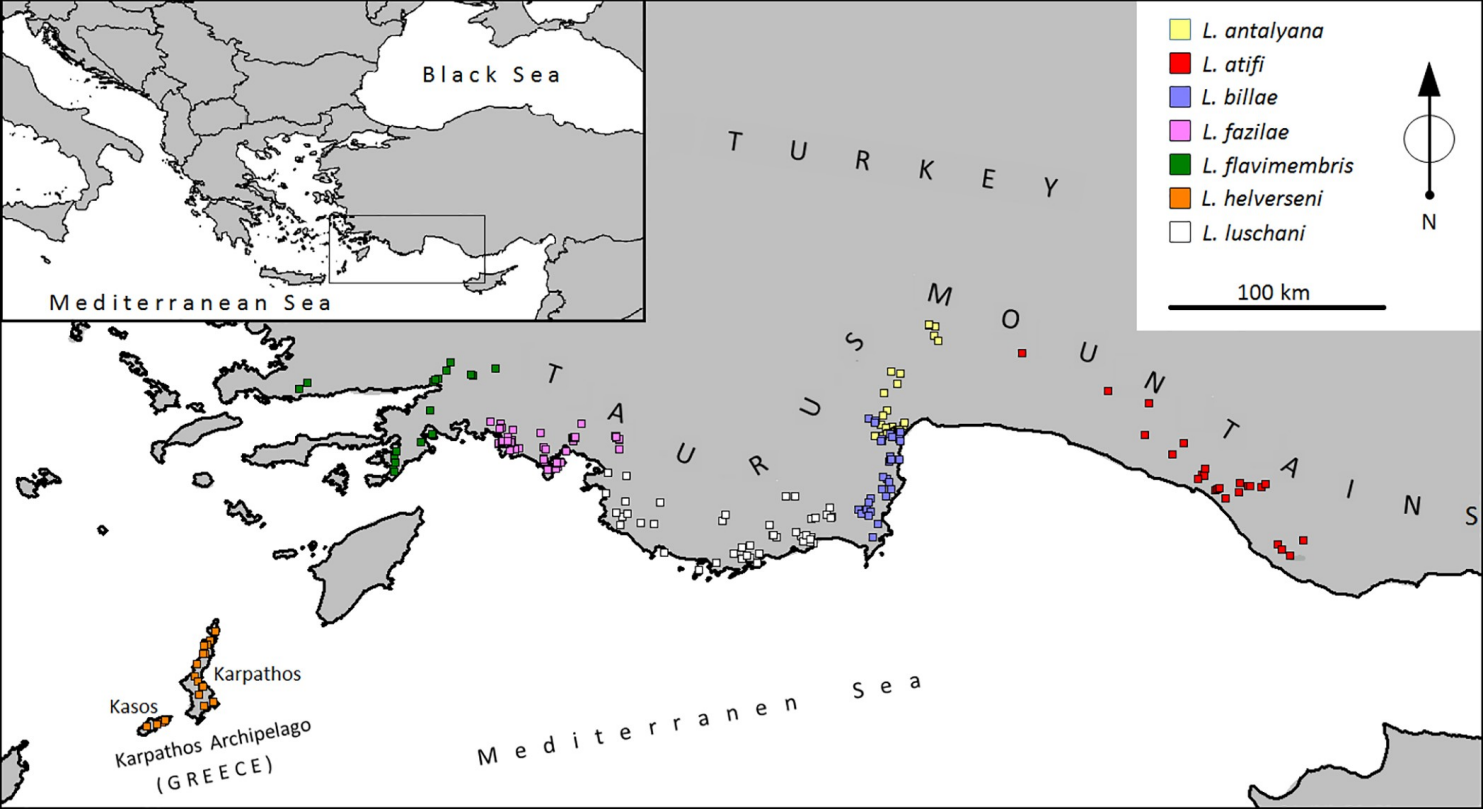
Localities along the Western Taurus Mountains and the Karpathos Archipelago in the eastern Mediterranean (see inset) of seven *Lyciasalamandra* species, used for Species Distribution Models (SDMs) and genetic analyses. For details see S1 File.

We analysed 559 specimens of *Lyciasalamandra* from 118 populations (population average 4.7 specimens) by either using sequences stored in GenBank or by analysing new tissue samples (Fig 1, S1 File and S1 Table). They cover all currently described species and subspecies as well as their entire ranges. Turkish samples were collected under license according to the ethical permission of the Ege University Animal Experiments Ethics Committee (2014#001) and special permission (2014#62406) for field studies from the Republic of Turkey, Ministry of Forestry and Water Affairs. Sampling in Greece took place according to the Hellenic National Law (Presidential Decree 67/81) and under a special permit (#107439/758) issued by the Ministry of the Environment. Salamanders were collected by turning stones in the respective habitats; tissue samples (toe clips) were immediately stored in absolute ethanol.

Due to the large number of species and populations studied, we throughout this study consistently use the following terms: first three digits (###) of a species’ name are followed by phyloclade (###-<Roman numeral>), population (###-p<Arabic numeral>) and haplotype (###-h<Arabic numeral>) codes.

### DNA extraction and sequencing

DNA was isolated using the Qiagen Blood and Tissue Kit following the manufacturer’s protocol. We sequenced fractions of two mitochondrial genes: 16SrDNA (primers 16SAL and 16SBH of [28]; initial melting for 120 s at 94°C, 33 cycles of denaturation for 30 s at 94°C, primer annealing for 30 s at 51°C, extension for 60 s at 65°C, final step at 65°C for 10 min) and ATPase (primers L-LYS-ML and H-COIII-ML of [8], covering fractions of subunits 8 and 6; initial melting for 10 s at 94°C, 30 cycles of denaturation for 30 s at 98°C, primer annealing for 30 s at 67°C, extension for 30 s at 72°C, final step at 72°C for 1 min). PCR reactions were prepared using either 5Prime Master Mix (16S) or the Phusion Flash Master Mix of Thermo Science (ATPase). PCR products were purified using the High Pure PCR Product Purification Kit of Roche. Sanger reactions for all genes were run using the Big Dye Terminator (ABI) with initial melting for 60 s at 96°C, 25 cycles of denaturation for 10 s at 96°C, primer annealing for 5 s at 50°C, extension for 240 s at 60°C. We sequenced single stranded fragments from both directions each on an ABI 3500 Genetic Analyzer Serie 2 automatic sequencer using standard protocols.

### Sequence alignment

We automatically aligned the single gene datasets (16S, ATP6 and ATP8) with MAFFT (version 7, [29]) using the iterative refinement [30, 31] and the Needleman-Wunsch algorithm [32]. We examined our datasets for stop codons and excluded them where necessary. Between base pair positions 244–251 we found an AT tandem repeat in several species; we had to exclude this section from further analyses since it produces additional local variation which could mask the true haplotype genealogy. All ambiguous areas in the alignment were excluded using GBlocks (version 0.91b, [33]). We used the package ape [34] and the function cbind in R (version 3.3.1, [35]) for sequence concatenation. We removed redundant sequences by collapsing them into haplotypes using FaBox [36].

Partition Finder (version 2.0.0, [37]) was used to select the best-fitting partitioning schemes and substitution models from a predefined number of subsets (one subset for 16S and one for each codon position in ATP6 and ATP8), implementing the greedy algorithm and the Akaike Information Criterion (AIC). We compared the Partition Finder runs with linked and unlinked branch length and selected the model with linked branch length across partitions according to the AIC value.

### Analyses of demographic population history

Nucleotide diversity (*π*) and haplotype diversity (*hd*) were calculated to estimate the genetic diversity of distinct phyloclades using DnaSP v. 6.10 [38] (for phyloclade delineation see below). To gain information about the demographic history of phyloclades we calculated further statistics: pairwise mismatch distribution [39], with observed distributions tested against expected distributions under a constant growth and a growth-decline model, respectively; Tajima’s *D* [40]; and Fu’s *Fs* [41]; all analyses done with DnaSP (see S1 Fig for mismatch distribution). The mismatch distribution describes the frequency of pairwise substitutional differences among individuals of a given group and is expected to be unimodal in populations that underwent recent bottleneck and rapid expansion [39]. Tajima’s *D* statistic tests for a departure from neutrality as measured by the difference between the number of segregating sites (*h*) and the average number of pairwise nucleotide differences (*p*). In the absence of balancing or purifying selection, population expansion can cause significant negative departures of Tajima’s *D* from zero, while a population bottleneck can cause a significant positive departure from zero [40]. Under the assumption of neutrality, Fu’s *Fs* statistic provides a test for population growth as well by identifying an excess of rare haplotypes in an expanding population when compared with the number of expected haplotypes in a stationary population (*Fs*<0; [41]). Conversely, *Fs*>0 would indicate a recent population bottleneck. *Fs-*values should be regarded significant if *p*<0.02 [41]. As additional test statistics for detecting patterns of population growth, we calculated *R*_*2*_ of [42], which has shown by these authors to be superior to the raggedness statistic *rg* of [43]. Statistical significance of *R*_*2*_ was tested using coalescent simulations in DnaSP. In addition, the demographic population history was reconstructed for each phyloclade using the Bayesian Skyline Plots (BSP) [44] method in Beast (version 2.5.0, [45]). We used the selected partitions of Partition Finder as predefined subsets and bModeltest for calculating the substitution models and the phylogeny in one single analysis [46]. As estimation for the substitution rate we inferred the mean rate of [6] (9.70E-3 sites per Mya) as strict clock rate. Coalescent Bayesian Skyline was used as tree prior and MCMC tests were run for 10 million steps each and sampled every 1,000^th^ generation. BSP can be found in S2 Fig.

### Analysis of molecular variance (AMOVA)

Ordered mitochondrial haplotypes (based on a p-distance matrix) were used to test for the degree of population fragmentation since, depending on their phylogenetic background, they may harbour information about their own history [47]. A hierarchical AMOVA [48] (ARLEQUIN software of [49]) partitioned the total genetic variance into variance components within populations (V_IP_), among populations within phyloclades (V_PC_) and among phyloclades within species (V_CS_). We also calculated the respective inbreeding coefficients (F-values) according to Weir and Cockerham [50]. Exact probabilities were calculated using the Markov-chain method [51], with 1023 recombinations and replicates each.

### Phylogenetic tree and haplotype networks

A phylogenetic tree of all haplotypes was calculated using Maximum Likelihood (ML) and Bayesian Inference (BI). For hierarchical outgroup rooting we added homologous gene fragments from complete mitochondrial genomes of *Salamandra salamandra* (EU880331), *Chioglossa lusitanica* (EU880308), *Mertensiella caucasica* (EU880319), *Pleurodeles poireti* (EU880329), *Pleurodeles waltl* (EU880330), *Euproctus platycephalus* (EU880317) and *Euproctus montanus* (EU880316). The ML tree was calculated with RAxML (Stamatakis 2014) using rapid bootstrapping and the greedy algorithm, running 2,000 bootstrap replicates. The BI tree was calculated with MrBayes (version 3.2.6, [52, 53, 54]) implementing two runs with four independent chains; each run with 10 million generations and sampled every 1,000^th^ generation with a burn-in of 20% (see S3 Fig for the BI tree). Afterwards we checked if the tree likelihood had converged. The results of Partition Finder were used as a priori configurations in MrBayes (see S2 Table). Since we could only use one substitution model for all partitions in RAxML [55], we decided to use the GTR+G model for all partitions.

We generated species specific population level genealogies using TCS [56]. TCS is widely used to calculate haplotype networks based on statistical parsimony. We set a probability cut-off value of 90% that defined the maximum number of mutational connections between pairs of sequences and thus delineated unconnected haplotype networks as distinct intraspecific phyloclades.

### Modelling of current and past species distributions

Grid-based correlative Species Distribution Models (SDMs; [57]) were used to predict the current potential distributions of *Lyciasalamandra* species within the general region of the genus’ known range. For this purpose, we compiled 253 geo-referenced localities of all species based on literature and on own fieldwork data (S1 File). The minimum number of records per species was 18 (details below) after elimination of duplicates in the same grid cell, applying the grid system of SDM climate data (see below). Although SDM building is delicate when the number of records is low (cf. [58]), it can be feasible in narrow-ranged species [59], which is why we continued to compute SDMs for all seven species.

As ecological predictors we used current high resolution climate data for the period 1979–2013 from the CHELSA project ([60]; version 1.2; data available at: http://chelsa-climate.org/, accessed 10 May 2018). CHELSA operates on monthly means and is based on a quasi-mechanistic statistical downscaling of the ERA interim global circulation model (GCM) with a GPCC bias correction [60]. The CHELSA website provides ‘bioclim’ variables (cf. [61]). From the 19 variables available, six were selected via pair-wise Pearson correlation analyses to avoid effects of multicollinearity, which is important when projecting SDMs into new space and time [62]. Of highly correlated variables (|r| > 0.7), we excluded the less informative one, based on a priori assumptions on biological importance to our target organisms: bio3, isothermality (BIO2/BIO7) (* 100); bio11, mean temperature of coldest quarter; bio15, precipitation seasonality (coefficient of variation); bio18, precipitation of warmest quarter; bio19, precipitation of coldest quarter.

CHELSA climate data are available at grid resolution 30 arc sec, thus reflecting macro- or mesoclimate. Like multiple authors before (cf. [57]), we consider these data as proxies to micro-climatic conditions. This is a crucial point to mention, especially with regard to *Lyciasalamandra* species, because part of their life these amphibians exploit deep-reaching systems of crevices in karstic limestone systems [3, 27, 63]. We defend our approach, as micro-climatic data for the focal taxa are not available and Rödder et al. [26] have shown that macro-climate can be effectively used for the computation of *Lyciasalamandra* SDMs. Along with these authors, we expect that CHELSA climate data show where the climate in general is favorable to our study species.

With the goal to predict the potential geographic ranges of the target species during the LGM (ca. 21 K BP), SDMs based on the current climate were projected to LGM data available from the CHELSA website (i.e., bioclim variables, downloaded 10 May 2018). They are based on the implementation of the CHELSA algorithm on PMIP3 (Paleoclimate Modelling Intercomparison Project Phase III) data using the widely used and well performing Community Climate System Model 4 global circulation model [64] as a test dataset and a paleo-digital elevation model with resolution 30 arc sec [60].

Maxent 3.4.1 [65, 66] was used for SDM building (https://biodiversityinformatics.amnh.org/open_source/maxent/, accessed 10 May 2018). This presence-only/background method operates with a machine-learning algorithm following the principle of maximum entropy. It makes predictions on the potential geographic range of a taxon by taking environmental (here: climatic) data from geo-referenced species records and random background data [65, 67]. In this way, it contrasts the environmental conditions at species’ presences against those at the background to fit a function to estimate the relative suitability to the species [68]. Maxent is a widely used SDM tool and often performs better than other SDM methods [69, 70]. It offers various settings for SDM building allowing fine-tuning [65, 70]. This requires some caution, however, as with these, the output can be dramatically altered when uncritically used [67, 68, 71, 72]. Therefore, it is important to explore settings and to adapt them to the available data [66, 70, 73].

In our final model runs, we employed Maxent with specifications for SDMs based on small sample sizes with hinge features. In three species (*L*. *antalyana*, *L*. *flavimembris*, *L*. *helverseni*), with 29, 18 and 23 records, respectively, the cross-validate approach was chosen. For all other species (with 33 or more records), the subsample approach was used with each 25% of the records randomly set aside as test data. The number of replicates was equal to the number of records in the cross-validate and 100 in the subsample approach. Extrapolation was used, but no clamping, and response curves were explored. One background was chosen for all species as a window enclosing all *Lyciasalamandra* records. The number of background points was 100,000. All other settings were default [66, 68, 70, 74]. A Multivariate Environmental Similarity Surfaces (MESS) analysis [71] revealed that there were no conditions in the general region of the genus’ known range (i.e. coastal mountain ranges and coastal islands) potentially leading to unrealistic extrapolations of response curves (S4 Fig).

Maxent calculates the area under the receiver operating characteristic curve (AUC) as a measure of predictive accuracy [65]. Following the classification of [75], AUC values range between 0.5 for models with no predictive ability and 1.0 for models giving perfect predictions; values > 0.9 describe ‘very good’, > 0.8 ‘good’, > 0.7 ‘useable’ models. Although criticized (e.g., [67]), the AUC is informative as it mirrors the model’s ability to distinguish between species records and background points, i.e. showing how general or restricted a distribution is along the range of the variables in the studied region [68]. To account for AUC critics, we also calculated True Skill Statistic (TSS) as sensitivity + specificity– 1 [76].

The Maxent default ClogLog output format (ranging 0–1) was chosen for processing the resulting SDM maps in a GIS approach, and the ‘maximum training sensitivity plus specificity ClogLog threshold’ was used to distinguish potential presence from absence, as this threshold might not overestimate distributions [66]. Because of the almost exclusive association of *Lyciasalamandra* species with karstic limestone formations (see above), areas identified with SDMs climatically suitable but outside these geological conditions were cut off using a geology shapefile of the Central Energy Resource Team of the US Geological Survey (http://energy.cr.usgs.gov/oilgas/wep/, accessed 10 May 2018). In addition, in LGM maps a sea level layer was overlaid to only show areas beyond today’s coast line that indeed were accessible continental shelf. It was obtained from the LGM Vegetation Download Page (http://anthro.unige.ch/lgmvegetation/download_page_js.htm, accessed 10 May 2018).

We prepared boxplots of Maxent ClogLog values at species records for current and LGM SDMs. These were tested using a Wilcoxon signed rank test.

## Results

### Molecular diversity and population demography

Altogether, we found 153 combined 16S and ATPase haplotypes, with the fewest in *L*. *flavimembris* and the most in *L*. *billae* (Table 1). The majority of haplotypes were private to a single population (78.4%). Only one haplotype occurred in a maximum of seven populations (ant-h1 of *L*. *antalyana*). Consequently, haplotype diversity was high in all phyloclades, with *hd* almost always approaching a maximum value of 1.0. In contrast, nucleotide diversity *π* was mostly below 1%.

**Table 1:** Genetic diversity, neutrality tests and mismatch goodness-of-fit tests for *Lyciasalamandra* phyloclades.

Phyloclade	*np*	*ns*	*nh* (*nph*) %*ph*	*hd* (s.d.)	*π* (s.d.)	Neutrality tests		Goodness-of-fit tests
						Tajima’s *D* (*P*-value)	Fu’s *Fs* (*P*-value)	*R*_*2*_ (*P*-value)
***L*. *atifi*-I (ati-I)**	3	18	4 (3) 75%	1.000 (0.177)	0.0028 (0.0009)	-0.79684 (> 0.10)	-1.514 (0.180)	0.2179 (0.164)
***L*. *atifi-*II (ati-II)**	10	52	14 (10) 71%	1.000 (0.027)	0.0067 (0.0006)	-0.16290 (> 0.10)	-9.421 (< 0.001)	0.1299 (0.374)
***L*. *atifi-*III (ati-III)**	3	14	3 (2) 67%	1.000 (0.272)	0.0028 (0.0009)	n.a.	n.a.	n.a.
***L*. *antalyana-*I (ant-I)**	8	36	11 (8) 72%	0.964 (0.051)	0.00431 (0.0042)	0.11338 (> 0.10)	-3.679 (0.021)	0.1413 (0.251)
***L*. *antalyana-*II (ant-II)**	8	30	3 (2) 67%	1.000 (0.272)	0.0022 (0.0008)	n.a.	n.a.	n.a.
***L*. *billae-*I (bil-I)**	7	29	8 (6) 75%	1.000 (0.063)	0.0653 (0.0009)	0.43162 (> 0.10)	-3.463 (0.030)	0.1665 (0.348)
***L*. *billae-*II (bil-II)**	1	13	2 (2) 100%	1.000 (0.500).	0.0011 (0.0006)	n.a.	n.a.	n.a.
***L*. *billae-*III (bil-III)**	9	35	14 (10) 71%	1.000 (0.027)	0.0064’ (0.0007)	0.41361 (> 0.10)	-9.990 (< 0.001)	0.1555 (0.451)
***L*. *billae-*IV (bil-IV)**	9	30	3 (0) 0%	1.000 (0.272)	0.0015 (0.0005)	n.a.	n.a.	n.a.
***L*. *luschani-*I (lus-I)**	4	14	4 (3) 75%	1.000 (0.177)	0.0048 (0.0013)	1.32331 (> 0.10)	-0.715 (0.328)	0.2486 (0.342)
***L*. *luschani-*II(lus-II)**	2	4	2 (2) 100%	1.000 (0.500)	0.0022 (0.0011)	n.a.	n.a.	n.a.
***L*. *luschani-*III (lus-III)**	1	6	3 (3) 100%	1.000 (0.272)	0.0015 (0.0005)	n.a.	n.a.	n.a.
***L*. *luschani-*IV (lus-IV)**	2	8	4 (4) 100%	1.000 (0.177)	0.0019 (0.0005)	0.00186 (> 0.10)	-2.181 (0.101)	0.2003 (0.059)
***L*. *luschani-*V (lus-V)**	9^c^	48	13 (11) 85%	1.000 (0.030	0.0055 (0.0006)	-0.18976 (> 0.10)	-9.501 (< 0.001)	0.1242 (0.199)
***L*. *luschani-*VI (lus-VI)**	7	30	7 (6) 86%	1.000 (0.076)	0.0021 (0.0003)	-1.12898 (> 0.10)	-5.925 (0.003)	0.0851 (<0.001)
***L*. *fazilae-*I (faz-I)**	9	34	8 (5) 63%	1.000 (0.063	0.0054 (0.0009)	0.21018 (> 0.10)	-4.022 (0.018)	0.1698 (0.352)
***L*. *fazilae-*II (faz-II)**	1	1	1 (1) 100%	n.a.	n.a.	n.a.	n.a.	n.a.
***L*. *fazilae-*III (faz-III)**	2	14	5 (5) 100%	1.000 (0.126)	0.0031 (0.0007)	-0.19092 (> 0.10)	-2.371 (0.085)	0.1599 (0.056)
***L*. *fazilae-*IV (faz-IV)**	6	27	10 (9) 90%	1.000 (0.045)	0.0030 (0.0007)	-1.62991 (> 0.05)	-8.843 (< 0.001)	0.0710’ (< 0.001)
***L*. *fazilae-*V (faz-V)**	2	8	3 (2) 67%	1.000 (0.272)	0.0015 (0.0005)	n.a.	n.a.	n.a.
***L*. *flavimembris* (fla-I)**	8	39	10 (7) 70%	1.000 (0.045	0.0047 (0.0005)	-0.36945 (> 0.10)	-6.650 (0.001)	0.1192 (0.046)
***L*. *helverseni-*I (hel-I)**	12	55	17 (15) 88%	0.985 (0.025)	0.0044 (0.0006)	-1.86018 (< 0.05)	-10.682 (< 0.001)	0.0675 (<0.001)
***L*. *helverseni-*II (hel-II)**	1	4	3 (3) 100%	1.000 (0.272)	0.0030 (0.0011)	n.a.	n.a.	n.a.
***L*. *helverseni-*III (hel-III)**	1	5	1 (1) 100%	n.a	n.a.	n.a.	n.a.	n.a.

*np*, number of populations; *ns*, number of samples; *nh* (*nph*) %*ph*, number of (private) haplotypes and percentage of private haplotypes; *π* (s.d.), nucleotide diversity with standard deviation; *hd* (s.d.), haplotype diversity with standard deviation; *R*_*2*_, Ramos-Onsins’ and Rozas’ [42] raggedness index; n.a., not applied.

**Fig 2:**
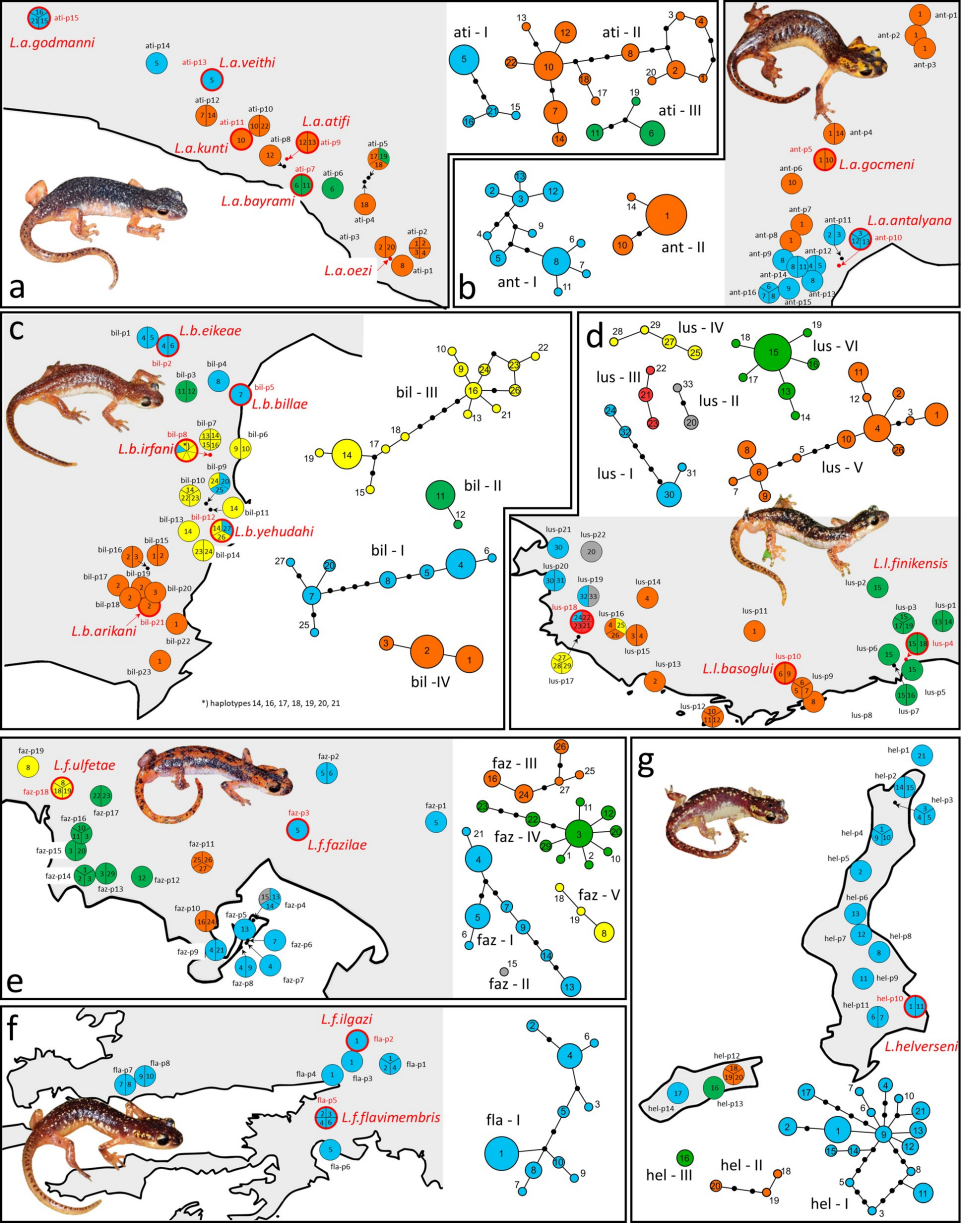
Geographic haplotype distribution and unrooted haplotype networks of Lycian salamander phyloclades. From East to West (see also Fig 1 for the distribution of all species): a) *L*. *atifi* phyloclades ati-I to -III, b) *L*. *antalyana* phyloclades ant-I and -II, c) *L*. *billae* phyloclades bil-I to -IV, d) *L*. *luschani* phyloclades lus-I to -VI, e) *L*. *fazilae* phyloclades faz-I to V, f) *L*. *flavimembris* (fla-I) and g) *L*. *helverseni* phyloclades hel-I to -III. Names and type localities of currently recognized subspecies are indicated in red in the distribution maps. Numbers in circles refer to found haplotypes, with circle size being proportional to haplotype abundance; black dots indicate haplotypes not found. Each line in between two haplotypes accounts for one mutational difference.

Analyses of population demography show in parts contradicting results within and among phyloclades (Fig 2, Table 1 and S1 Fig). Bayesian skyline plots indicate a recent increase in effective population size for ant-I, ant-II, bil-III, lus-VI, faz-IV and fla-I, while a recent decrease is indicated for ati-III, bil-I, lus-V, faz-III and, most pronounced, for hel-I (S2 Fig). Interestingly, for the latter significant negative values of Tajima’s *D* and Fu’s *Fs* indicate possible recent population expansion. In all other phyloclades, according to Bayesian skyline plots population sizes remained more or less constant (S2 Fig), although in several cases significantly negative values of Fu’s *Fs* indicate possible recent population expansion, while Tajima’s *D* did not significantly deviate from zero. In contrast, significantly negative values of Fu’s *Fs* (*p*<0.02) indicate possible recent population expansion in ati-II, bil-III, lus-V, lus-VI, faz-I, faz-IV, fla-I and hel-I, while the same is indicated by a significantly negative value of Tajima’s *D* only for hel-I. The graphic representation of the mismatch distribution tentatively fits an expected growth-decline model only in phyloclades ant-II, lus-IV, lus-VI, and hel-I. In all other phyloclades, the observed mismatch distribution seems to fit neither a growth decline nor a constant growth model (S1 Fig). *R*_*2*_ values significantly different from a neutral model were only found for lus-VI, faz-IV, fla-I and hel-I (Table 1). For all other phyloclades, multimodal mismatch distributions indicate a pattern of recurrent population bottlenecks and expansions.

Total molecular variance was lowest in *L*. *atifi* (6.9%) and highest in *L*. *fazilae* 19.8 (Table 2). Molecular variance distribution shows a consistent pattern across species. Within-population variance was extremely low, ranging from 1.50% in *L*. *luschani* to 7.89% in *L*. *billae*. The respective fixation indices F_IP_ were close to one, indicating almost fixed molecular differences among populations. Among phyloclade molecular variance made up most of the entire molecular variance, with a maximum of 91.5% in *L*. *fazilae* (a comparable three-level AMOVA could not be calculated for *L*. *flavimembris* since only one phyloclade was found in this species).

**Table 2:** Intraspecific distribution of molecular haplotype variance (AMOVA).

	among phyloclades					among populations within phyloclades					within populations					total variance	
species	df	V_CS_	%V_CS_	F_CS_	p	df	V_PC_	%V_PC_	F_PC_	p	df	V_IP_	%V_IP_	F_IP_	p	df	V_T_
***L*. *atifi***	2	4.810	69.56	0.696	< 0.001	13	1.748	25.80	0.847	< 0.001	68	0.321	4.64	0.953	< 0.001	83	6.915
***L*. *antalyana***	1	6.270	83.59	0.836	< 0.001	14	1.002	13.36	0.814	< 0.001	52	0.229	3.05	0.969	< 0.001	67	7.051
***L*. *billae***	3	6.651	79.17	0.791	< 0.001	22	1.087	12.94	0.621	< 0.001	81	0.663	7.89	0.921	< 0.001	106	8.401
***L*. *luschani***	5	16.447	91.18	0.912	< 0.001	19	1.321	7.32	0.830	< 0.001	87	0.271	1.50	0.985	< 0.001	111	18.039
***L*. *fazilae***	4	18.090	91.50	0.915	< 0.001	15	1.341	6.78	0.798	< 0.001	64	0.340	1.72	0.983	< 0.001	83	19.771
***L*. *helverseni***	2	15.548	87.26	0.873	< 0.001	11	1.703	9.56	0.750	< 0.001	63	0.567	3.18	0.968	< 0.001	63	17.818

Among phyloclades (V_CS_), among populations within phyloclades (V_PC_), within populations (V_IP_); the respective fixation indices F_CS_, F_PC_ and F_IP_ are given. Not calculated for *L*. *flavimembris* due to the lack of an intraspecific phyloclade sub-structure.

### Haplotype networks and spatial haplotype distribution

Two to six distinct phyloclades appeared in all species but *L*. *flavimembris* (only one phyloclade; Fig 2A–2G). Most phyloclades correspond to distinct geographic clusters. Exceptions are ati-II (interspersed by ati-III), bil-I (some haplotypes also occur throughout the range of bil-III) and hel-I (it occurs on all islands of the Karpathos Archipelago, but with hel-II and–III being found in between its populations). Within most phyloclades there seems to exist a fine-scale geographic differentiation that is largely mirrored by haplotype positions within the networks, e.g. in *L*. *atifi* (ati-I, ati-III), *L*. *antalyana* (ant-I, ant-II), *L*. *billae* (buil-I, bil-IV), *L*. *luschani*, *L*. *fazilae* and *L*. *flavimembris*. Discordant network and geographic positions of haplotypes or groups of haplotypes are obvious in *L*. *atifi* (ati-II), *L*. *billae* (bil-III) and, to a lower degree, in *L*. *helverseni* (hel-I). Widespread haplotypes spanning almost the entire range of a phyloclade only occur in *L*. *antalyana* (ant-h1), *L*. *billae* (bil-h1) and *L*. *luschani* (lus-h15). Haplotypes of two phyloclades co-occur in *L*. *atifi* (ati-p5), *L*. *billae* (bil-p8, bil-p9, bil-p12), *L*. *luschani* (lus-p16, lus-p18, lus-p19) and *L*. *fazilae* (faz-p4)

### Modelled current and past potential species distributions

AUC values of SDMs ranged from 0.854 to 0.9997, suggesting ‘good’ to ‘very good’ model performance. This was also supported by TSS values ranging 0.762 to 0.879. Acceptable model accuracy was also indicated by the markedly low standard deviation of single model runs (ClogLog ≤ 0.2). The modelled potential distributions of the species under current climate (S5 Fig) well explained most known species’ presences and identified more or less continuous areas as suitable within the proximity of the species’ records and beyond. However, in *L*. *atifi*, *L*. *billae*, *L*. *fazilae* and *L*. *luschani* some records remained unexplained under the ‘maximum training sensitivity plus specificity ClogLog threshold.

**Fig 3:**
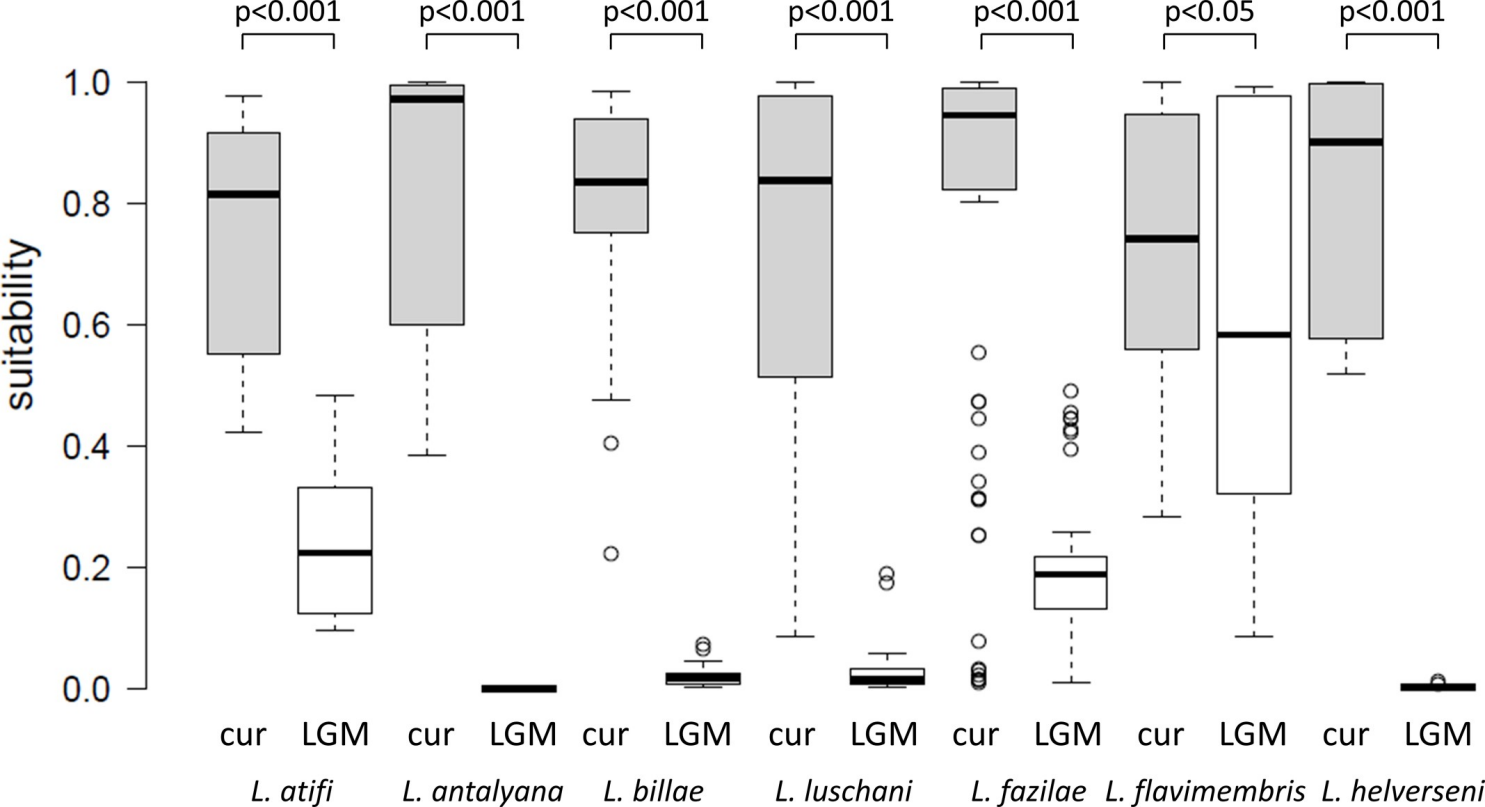
Box-Whisker plots for Maxent ClogLog values at known records of *Lyciasalamandra* species under current (grey) and LGM (white) climatic conditions. Values range from 0 (no suitability) to 1 (perfect suitability).

When projecting SDMs into climatic conditions of the LGM, species’ potential distributions markedly shifted (*L*. *fazilae*, *L*. *flavimembris*, *L*. *helverseni*), decreased (*L*. *atifi*, *L*. *luschani*) or even no suitable area could be identified (*L*. *antalyana*, *L*. *billae*) (S5 Fig). In all taxa, it became evident that all or almost all of today’s known records had been in unsuitable areas during the LGM. The suitability alteration in terms of Maxent ClogLog values at species records is illustrated in Fig 3. The median was highly significantly different in all species (*p* < 0.001), except in *L*. *flavimembris* (*p* = 0.041), when comparing current and LGM climates.

## Discussion

### Phylogeographic structure of species

Lycian salamanders have inhabited the Anatolian Mediterranean coast and the Karpathos Archipelago for more than 10 mya [6]. Subsequent diversification resulted in the seven species known today, with a varying number of distinct intraspecific lineages (i.e. some suggested to be subspecies). Several authors had already emphasized that *Lyciasalamandra* populations often harbour exclusive haplotypes, indicating a high degree of isolation among populations (e.g., [6, 8, 77]); however, their sampling was geographically more limited. Our dense spatial sampling of 118 populations across the entire known geographic range of the genus, combined with new analytic approaches, now offers the opportunity to evaluate the degree of possible isolation among populations as well as the processes that shaped this unique pattern in this enigmatic amphibian clade.

All but one (*L*. *flavimembris*) species show a pattern of deep haplotype differentiation in the mtDNA with pronounced geographic structure, indicating possible long-term isolation and/or limited dispersal and gene flow capabilities [78; currently, the alternative hypothesis of male-biased gene flow, which would erode population differentiation, cannot be ruled out due to the fact that bi-parentally inherited nuclear genes have not yet been investigated]. These highly divergent allopatric phyloclades show almost no sign of geographic overlap, with the shortest distance between two phyloclades being 2.5 km in *L*. *helverseni* and the longest being 22 km in *L*. *luschani*. Within most phyloclades, further well-differentiated haplogroups exist, which again commonly are allopatric (shallow haplotype trees showing geographic structure; [78]). Given the high degree of differentiation among phyloclades and their small-scale geographic occurrence, we consider this one of the most exceptional cases of fine-scale genetic differentiation in mtDNA within an amphibian. Of course, comparative levels of intraspecific differentiation at mitochondrial loci were described also in other amphibian species. European examples include *Rana temporaria* [79, 80], *Rana iberica* [81], *Hyla orientalis* [82], *Lissotriton boscai* [83], *Lissotriton helveticus* [84] and *Salamandra algira* [85]. However, samples of these species came from ranges of up to two orders of magnitude larger than those inhabited by *Lyciasalamandra* species (maximum distance between two samples of a species range from 40 km in *L*. *flavimembris* to 160 km in *L*. *atifi*, respectively). In addition, admixture of haplotypes of neighbouring phyloclades as observed in other species (e.g. *R*. *temporaria*; [80]), a pattern that often mirrors geographic introgression after secondary contact of lineages (in Europe most often after re-expansion from glacial refugia; [86, 87]), is found only in eight out of the 118 populations studied. Pronounced intraspecific phylogenetic discontinuity visible through high mitochondrial haplotype differentiation is typical for amphibians, presumably because their relative dispersal ability is low [88]. A complex population differentiation comparable to what we found in *Lyciasalamandra*, is also observed in some tropical species, mainly driven by forest habitats and topographic heterogeneity (e.g., [89]). However, in such cases haplotypes are often shared among populations (e.g., [90]).

### Phylogeographic structure within phyloclades

In all *Lyciasalamandra* species, even geographically nearby populations harbour private haplotypes, although, from a human point of view, the habitat in-between seems to be appropriate (most often karstic limestone with pine forests [3, 63]). This is most obvious in phyloclades ati-I of *L*. *atifi*, ant-I of *L*. *antalyana*, bil-I of *L*. *billae*, lus-I and lus-II of *L*. *luschani*, faz-II of *L*. *fazilae* and hel-I of *L*. *helverseni* (own field observation). From a geographic point of view, an extreme example is the island of Kasos, where all three phyloclades of *L*. *helverseni* occur within only 7.3 km (populations hel-p12, -13 and -14 in Fig 2F). Given the large mutational difference of such neighbouring haplotypes, a pattern like this is most easily be explained by strict isolation. Widespread haplotypes, covering a maximum area of 10 to 15 km in *Lyciasalamandra*, are rare and indicate either moderate recent female dispersal or incomplete lineage sorting. The most common haplotype in terms of populations (ant-h1 was found in seven populations) covers a distance of more than 50 km.

Widespread haplotypes are possibly indicative of geographic expansion, a process that is usually associated with demographic expansion (e.g., [88, 91]). Therefore, one would expect respective population genetic signals within phyloclades. This is in fact the case in ant-II and lus-VI, both having wide-spread haplotypes and both showing a signal of recent demographic expansion in the Bayesian skyline plots (S4 Fig). On the other hand, also some phyloclades with no widespread haplotypes showed signs of recent demographic expansion after a bottleneck (Table 1: significantly negative values of Fu’s *Fs*; alternatively, a selective sweep of mtDNA haplotypes may also produce such a pattern; however, this invokes pronounced female dispersal, which contradicts current knowledge of the ecology of *Lyciasalamandra* [4]). Only in hel-I this was corroborated by a significantly negative Tajima’s *D* (hel-I). Bayesian skyline plots in most cases showed only moderate signs of change in effective population sizes towards the recent (again hel-I is an exception). We therefore consider the calculated population genetic parameters to be less conclusive, which might be attributed to the fact that most phylogroups harboured predominantly haplotypes private to single populations.

Klewen [63] suggested that rivers and rock formations other than limestone act as strong barriers to dispersal and hence gene flow among *Lyciasalamandra* populations. However, in between such river systems, dispersal should have easily been possible, given that most often karstic limestone connects even adjacent populations. In fact, star-like haplotype networks with locally occurring descendent haplotypes or haplogroups branching off from a central one occur in several *Lyciasalamandra* phylogroups. Such patterns are typical of range expansions, followed by local differentiation and subsequent isolation [88, 91]. One reason why in most cases this pattern is not identified by population demographic parameters may be that such star-like structures almost always form only part of the respective haplotype network, with longer branches also connecting more differentiated haplotypes to the central haplotype. This inevitably increases the raggedness of mismatch distributions and increases deviation from a pattern expected under a simple growth-decline model. Therefore, and apart from the idea of strong isolation of populations, we conclude that in fact range expansion occasionally must have occurred regularly in the past. Future research including bi-parentally inherited genes is needed to test this assumption.

Interestingly, within phyloclade hel-I of *L*. *helverseni*, haplotype hel-h1 occurs at two localities, Olympos village (hel-p4) and the city of Pigadia (hel-p10) (see Fig 2F) at the almost respective ends of the island of Karpathos, ca. 27 km away from each other. Pigadia is the only harbour connecting Karpathos Island with the Greek mainland. We therefore assume that hel-h1 may be a case of anthropogenic translocation, in this case from Olympos village to the city of Pigadia (see review on distribution patterns of Aegean herpetofauna and examples of human mediated translocations therein [20]). Admittedly, incomplete lineage sorting, which may also produce a pattern of disjunctive haplotype distribution throughout a species’ distribution range by preserving ancestral polymorphisms cannot completely be ruled out as an alternative explanation, although in none of the other *Lyciasalamandra* phyloclades a similar pattern was observed.

### Potential range dynamics during the Quaternary

To explain the pattern of haplotype differentiation and distribution observed today, there must have been geological episodes when female dispersal was possible. According to [6], the Messinian Salinity Crisis (MSC) was most likely responsible for the onset of intraspecific evolution in *L*. *fazilae*, *L*. *helverseni* and *L*. *luschani*. In *L*. *atifi*, *L*. *antalyana*, *L*. *billae* and *L*. *flavimembris*, intraspecific diversification seems to have started in the Quaternary, a period when recurrent and dramatic climatic alterations [18, 22, 92] seem to have shaped the evolution of western Palearctic biota (e.g., [93, 94]). Unfortunately, the number of stitials and interstitials that occurred in the Quaternary is high [22]; so, given the uncertainty associated with split time estimation based on the molecular clock it is not possible to affiliate any split within this period to a single climatic event.

During the LGM, sites where nowadays *Lyciasalamandra* species occur were largely not explained (S5 Fig). At these sites, Maxent ClogLog values were significantly lower compared to current climate (Fig 3), strongly supporting that unsuitable conditions for salamander survival prevailed except for *L*. *fazilae* and *L*. *flavimembris*. In some species, SDM projections into the LGM revealed suitable area not too far from nowadays occurrences, all in all including areas which contain karstic limestone, too (S5 Fig). In part, e.g. in *L*. *helverseni*, this included the shelf area of the Mediterranean Sea which was about 100 m lower than today (e.g., [95]). In consequence, one may argue that range shifts in order to following suitable habitats could have occurred. There exist numerous examples of European plant and animal species for the validity of the so-called expansion-contraction (EC) model, a demographic scenario in temperate species whereby glacial cycles result in the contraction in size and shift toward lower latitudes into refugial areas during periods of cooling, followed by population growth and re-colonisation during postglacial warming (e.g., [86, 96]). Interestingly, numerous *L*. *fazilae* populations, especially those living far from the Mediterranean coast, appear to live under suboptimal current macroclimatic conditions (Fig 3, S5 Fig). This can be interpreted as an indication that horizontal range shifts due to climate change do not always allow theses salamanders to quickly adapt their geographic ranges to the climate.

Also not in line with such a past range shift scenario is our observation that almost no suitable area for four species, *L*. *antalyana*, *L*. *billae*, *L*. *luschani* and *L*. *helverseni*, could not be identified during the LGM (Fig 3, S5 Fig). This casts some doubt on potential range shift scenarios. Also, range shifts are typical bottleneck events and should leave characteristic imprints in the genetic signature of populations [87, 88, 93]. In the species where this has been observed, they led to an erosion of genetic diversity within and among populations, and after re-expansion genetically uniform populations would inhabited large areas [88, 93]. Species with a low dispersal capacity were ultimately doomed to extinction through loss of suitable habitat([97], unless they could shift their ranges at small scales along altitudinal gradients [93]. Alternatively, they might have survived in so-called refugial sanctuaries [84]. Any recurrent range shift, either on larger or on smaller geographic scales, would produce the above mentioned pattern of genetically similar and at the same time depauperate populations. We should observe such a pattern if *Lyciasalamandra* populations had followed shifting suitable habitats between stitials and interstadials and *vice versa*. Our results of numerous local haplotypes across most phyloclades’ ranges contrast with this expectation. Hence, micro-refugial survival in glacial sanctuaries best explains this unique pattern of fine-scale geographic allopatry of mitochondrial haplotypes within *Lyciasalamandra* (see [23, 98] for a further examples of cryptic diversity that evolved in the same area during Plio-Pleistocene glaciations).

Based on demographic and growth pattern analyses, [27] recently showed that the mainly subterranean life-style of Lycian salamanders in deep-reaching systems of crevices allows them to survive both cold winter and dry and hot summer. They observed a very small age- and size-related life-history variation across populations and species and concluded that this hints at a pronounced niche conservatism in *Lyciasalamandra*. Transferring this into a temporal dimension, this highly specialised and at the same time conserved ecological niche in combination with viviparity provides a sufficient explanation why many populations could have survived repeated climatic deteriorations ‘on the spot’. A pronounced female philopatry, which is considered advantageous when living in harsh environments as it can help females to acquire and defend an appropriate shelter (see [99] for the viviparous alpine salamander), may have enforced this niche conservatism.

### Did our mitochondrial markers capture the whole story?

Mitochondrial markers as studied by us are inherited almost exclusively by females [100], so gene flow mediated by males would remain undetected when studying organelle genes. Slight male-biased dispersal was suggested by [4] to explain discordant patterns of mitochondrial versus nuclear genes in a hybrid zone of *L*. *antalyana* and *L*. *billae*. Such gene flow, if strong enough, should admix nuclear alleles among populations, while population specific mitochondrial haplotypes would persist. However, several authors mentioned that neighbouring populations often show divergent, but within populations seemingly stable, phenotypes in terms of coloration and pattern (e.g., [3, 63]). Unfortunately, such studies neither were based on statistically solid sample sizes, nor did they appreciate the potentially high environmental and ontogenetic plasticity in amphibian colouration (e.g., [101, 102]). Genes for coloration and pattern are coded by the nuclear genome, and unless strong natural selection would locally stabilize distinct colour and pattern morphs, even occasional male-mediated gene flow would homogenize populations in terms of nuclear genes. Therefore, our observed pattern of strong isolation among populations does not seem to be an artefact of a merely mitochondrial based analysis. However, analyses of bi-parentally inherited nuclear genes are needed to test this hypothesis.

### Taxonomic implications

Within *Lyciasalamandra*, numerous new taxa have been described during the last few years, however, almost always based on morphology alone (see above). Our phyloclade structure and subspecies designation only match perfectly in *L*. *antalyana*, which is also supported by the phylogenetic tree. In *L*. *fazilae* and *L*. *helverseni*, phyloclades diversity is higher than current taxonomic diversity. In the phylogenetic tree, the five phyloclades of *L*. *fazilae* form three well supported clusters ((faz-I + faz-II), faz-II and (faz-IV + faz-V)), while in *L*. *helverseni*, phyloclade structure perfectly matches the three clusters supported by the pylogenetic tree.

In *L*. *atifi*, *L*. *billae* and *L*. *flavimembris* several subspecies have been described even within phyloclades that have formed in our analysis. Most extremely, in *L*. *billae* populations morphologically and geographically assigned to three subspecies, *L*. *b*. *billae* (bil-p6), *L*. *b*. *irfani* (bil-p7, -8) and *L*. *b*. *yehudahi* (bil-p9 to -14), respectively, even harbour haplotypes of the same phyloclades. Such patterns may be due to incomplete lineage sorting or secondary contact. *L*. *billae*, phyloclades bil-I, bil-II and bil-III form a common cluster, however, without three clearly delineated sub-clusters. The situation is similar in *L*. *atifi*: although ati-I and ati-III are well supported by the phylogenetic tree (S3 Fig), phyloclade differentiation is not mirrored by cluster delineation. *L*. *flavimembris* does not show any significant intraspecific differentiation, although two subspecies have been discriminated based on morphology alone. In *L*. *luschani*, clusters formed by the phylogenetic tree perfectly match current subspecies taxonomy, while the four phyloclades identified within *L*. *l*. *luschani* do not transfer to an unambiguous cluster pattern in the phylogenetic tree.

Variance distribution among phyloclades is more than twice as high in *L*. *luschani*, *L*. *fazilae* and *L*. *helverseni* compared to the other species (Table 2), indicating that also taxonomic differentiation within these species might be more justified. Almost all taxa newly described during the last decade were delineated exclusively on the basis of colour and pattern polymorphisms (e.g., [10–13]) and allopatric distribution. While the latter criterion is concordant with the definition of subspecies [103], the discriminant power of the morphological characters used for the delimitation of recently described taxa within *Lyciasalamandra* has never been proven. Nevertheless, and although we doubt the validity of the current taxonomy within *Lyciasalamandra*, we abstain from a taxonomic revision since our result are based on mitochondrial data alone.

## Supporting information

S1 Fig Comparison of observed mismatch distributions (obs) of *Lyciasalamandra* phyloclades with distributions expected under a growth-decline (exp (G-D)) and a constant growth (exp (cG)) model; X-axis: number of base substitutions between two haplotypes; Y-axis: frequency of the number of base substitutions between two haplotypes.
(JPG)

S2 Fig Bayesian Skyline Plots (BSP) for phyloclades of Lycian salamanders (a-f): a) *L*. *atifi* BSPs for phyloclades ati-I, ati-II and ati-III, b) *L*. *antalyana* BSPs for phyloclades ant-I and ant-II, c) *L*. *billae* BSPs for phyloclades bil-I and bil-III, d) *L*. *luschani* BSPs for phyloclades lus-I, lus-IV, lus-V and lus-VI, e) *L*. *fazilae* BSPs for phyloclades faz-I, faz-III and faz-IV, f) *L*. *flavimembris* BSP for phyloclade fla-I, and g) *L*. *helverseni* BSP for phyloclade hel-I. Phyloclades were coloured according to the colours of the haplotype networks in Fig 2.
(TIF)

S3 Fig Bayesian Inference (BI) tree of all haplotypes. Outgroups were excluded from the figure. We only represent Bayesian Posterior Probabilities ≥0.95 (see numbers at nodes). Phyloclades of each species were colour marked according to Fig 2 in the main text. Due to the low resolution of the ML tree, we only represent the BI tree.
(TIF)

S4 Fig Mapped result of the Multivariate Environmental Similarity Surfaces (MESS) analysis. (TIF)

S5 Fig Mapped Maxent SDMs for all *Lyciasalamandra* species under current (left) and LGM (right) climatic conditions. Note that under the LGM land masses exceeded those of today due to sea level change. Suitability to species increases with higher ClogLog values, indicated by warmer colours. Known species records are indicated by blue dots (cf. S1 File). For details of modelling and mapping see Materials and Methods.
(TIF)

S1 File Sampling localities used for SDMs and genetic analyses of seven *Lyciasalamandra* species. (XLSX)

S1 Table GenBank accession numbers of *Lyciasalamandra* haplotypes. (DOCX)

S2 Table Results of Partition Finder with partitions and substitution models (cp = codon position). (DOCX)
