# Supplementary figures and images for: Phylogeographic analyses point to long-term survival on the spot in micro-endemic Lycian salamanders
Source: PLoS One. 2020 Jan 13;15(1):e0226326. doi: 10.1371/journal.pone.0226326 (PMC6957296; doi:10.1371/journal.pone.0226326)

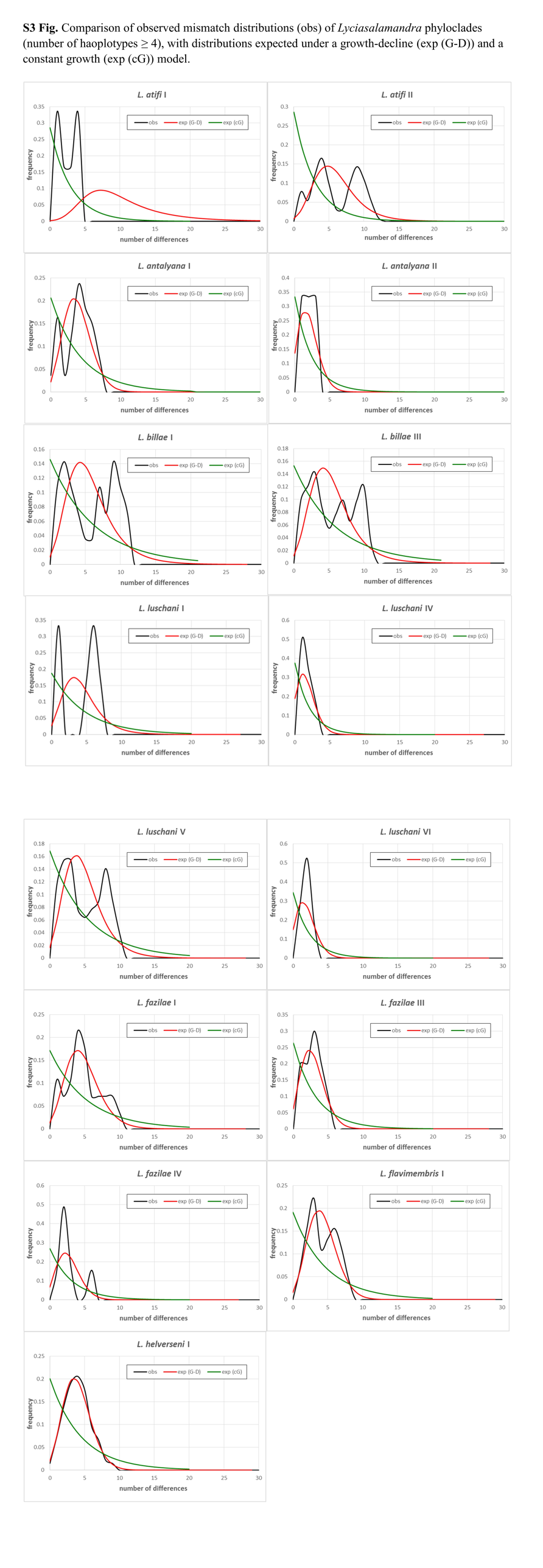

Supplement: S1 Fig — Comparison of observed mismatch distributions (obs) of Lyciasalamandra phyloclades with distributions expected under a growth-decline (exp (G-D)) and a constant growth (exp (cG)) model; X-axis: number of base substitutions between two haplotypes; Y-axis: frequency of the number of base substitutions between two haplotypes. (JPG) [file pone.0226326.s001.jpg]

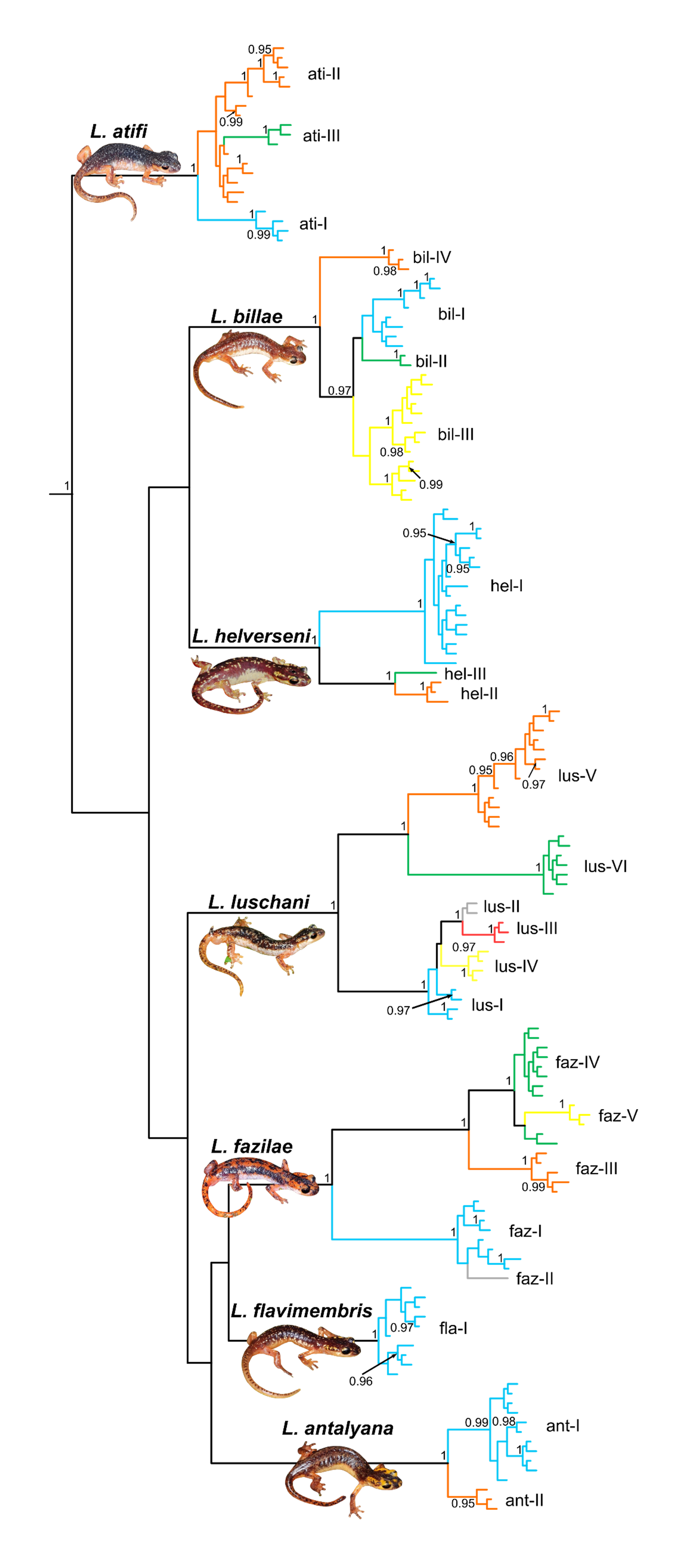

Supplement: S3 Fig — Outgroups were excluded from the figure. We only represent Bayesian Posterior Probabilities ≥0.95 (see numbers at nodes). Phyloclades of each species were colour marked according to Fig 2 in the main text. Due to the low resolution of the ML tree, we only represent the BI tree. (TIF) [file pone.0226326.s003.tif]

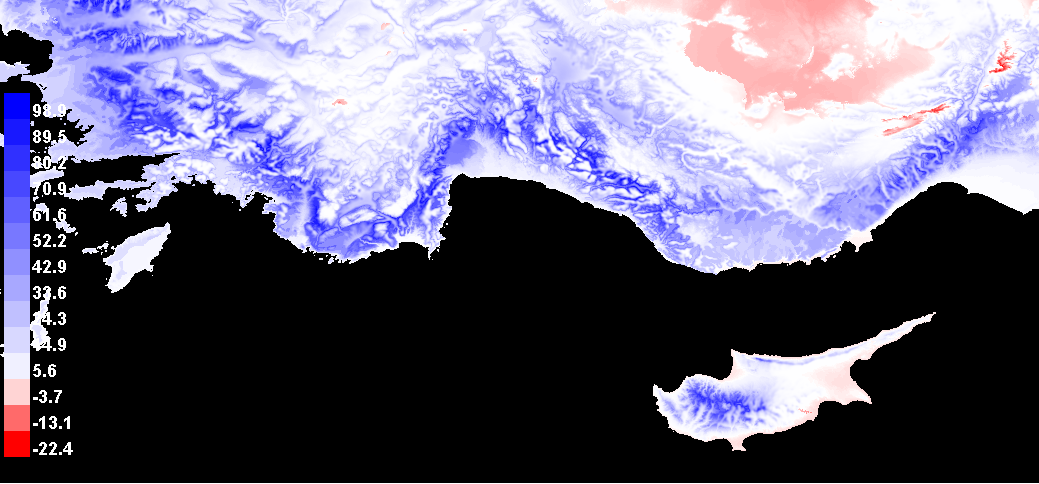

Supplement: S4 Fig — (TIF) [file pone.0226326.s004.tif]

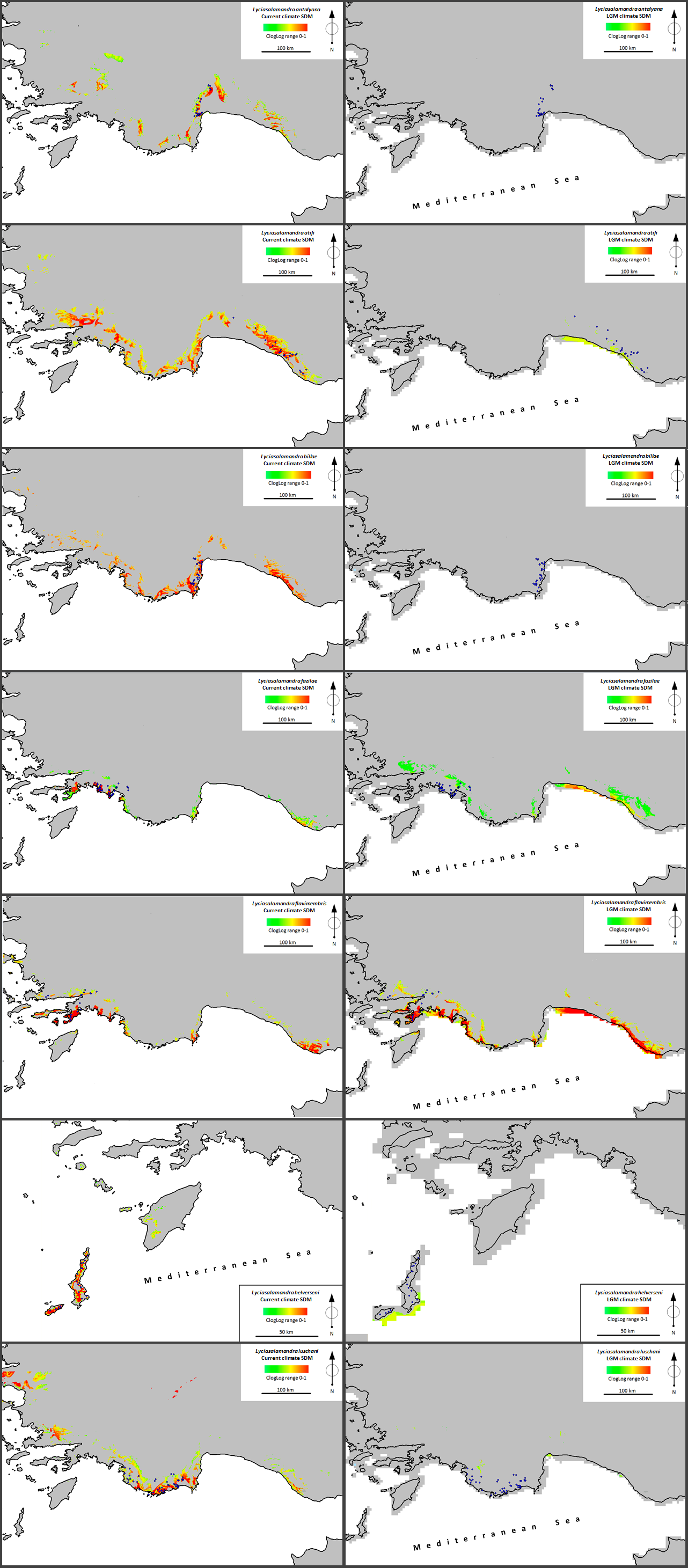

Supplement: S5 Fig — Mapped Maxent SDMs for all Lyciasalamandra species under current (left) and LGM (right) climatic conditions. Note that under the LGM land masses exceeded those of today due to sea level change. Suitability to species increases with higher ClogLog values, indicated by warmer colours. Known species records are indicated by blue dots (cf. S1 File). For details of modelling and mapping see Materials and Methods. (TIF) [file pone.0226326.s005.tif]
