# Supplementary material for: Phylogeographic analyses point to long-term survival on the spot in micro-endemic Lycian salamanders
Source: PLoS One. 2020 Jan 13;15(1):e0226326. doi: 10.1371/journal.pone.0226326 (PMC6957296; doi:10.1371/journal.pone.0226326)
Supplement: S1 Table — (DOCX) [file pone.0226326.s007.docx]

**S1 Tab.** GenBank accession numbers of *Lyciasalamandra* haplotypes.

| **taxon** | **haplotype** | **16S  (this paper)** | **ATP8  (this paper)** | **ATP6  (this paper)** | **16S  (Veith et al. 2008)** | **ATP8+6  (Veith et al. 2008)** |
| --- | --- | --- | --- | --- | --- | --- |
| *Lyciasalamandra antalyana* | H6 | **MN854712** | **MN867039** | **MN867162** |  |  |
| *Lyciasalamandra antalyana* | H7 | **MN854713** | **MN867040** | **MN867163** |  |  |
| *Lyciasalamandra antalyana* | H8 | **MN854714** | **MN867041** | **MN867164** |  |  |
| *Lyciasalamandra antalyana* | H1 | **MN854707** | **MN867034** | **MN867157** |  |  |
| *Lyciasalamandra antalyana* | H10 |  |  |  | **EU430956** | **EU430913** |
| *Lyciasalamandra antalyana* | H14 | **MN854717** | **MN867044** | **MN867167** |  |  |
| *Lyciasalamandra antalyana* | H8 |  |  |  | **EU430957** | **EU430915** |
| *Lyciasalamandra antalyana* | H11 |  |  |  | **EU430957** | **EU430914** |
| *Lyciasalamandra antalyana* | H12 |  |  |  | **EU430958** | **EU430916** |
| *Lyciasalamandra antalyana* | H3 | **MN854709** | **MN867036** | **MN867159** |  |  |
| *Lyciasalamandra antalyana* | H13 | **MN854716** | **MN867042** | **MN867166** |  |  |
| *Lyciasalamandra antalyana* | H9 | **MN854715** | **MN867042** | **MN867165** |  |  |
| *Lyciasalamandra antalyana* | H2 | **MN854708** | **MN867035** | **MN867158** |  |  |
| *Lyciasalamandra antalyana* | H4 | **MN854710** | **MN867037** | **MN867160** |  |  |
| *Lyciasalamandra antalyana* | H5 | **MN854711** | **MN867038** | **MN867161** |  |  |
| *Lyciasalamandra billae* | H1 | **MN854807** | **MN867134** | **MN867257** |  |  |
| *Lyciasalamandra billae* | H10 |  |  |  | **EU430969** | **EU430932** |
| *Lyciasalamandra billae* | H11 | **MN854813** | **MN867141** | **MN867264** |  |  |
| *Lyciasalamandra billae* | H12 | **MN854814** | **MN867140** | **MN867263** |  |  |
| *Lyciasalamandra billae* | H13 | **MN854815** | **MN867142** | **MN867265** |  |  |
| *Lyciasalamandra billae* | H14 | **MN854816** | **MN867143** | **MN867266** |  |  |
| *Lyciasalamandra billae* | H15 | **MN854817** | **MN867144** | **MN867267** |  |  |
| *Lyciasalamandra billae* | H16 | **MN854818** | **MN867145** | **MN867268** |  |  |
| *Lyciasalamandra billae* | H17 | **MN854819** | **MN867146** | **MN867269** |  |  |
| *Lyciasalamandra billae* | H18 | **MN854820** | **MN867147** | **MN867270** |  |  |
| *Lyciasalamandra billae* | H19 | **MN854821** | **MN867148** | **MN867271** |  |  |
| *Lyciasalamandra billae* | H2 | **MN854808** | **MN867135** | **MN867258** |  |  |
| *Lyciasalamandra billae* | H20 | **MN854822** | **MN867149** | **MN867272** |  |  |
| *Lyciasalamandra billae* | H21 | **MN854823** | **MN867150** | **MN867273** |  |  |
| *Lyciasalamandra billae* | H22 | **MN854824** | **MN867151** | **MN867274** |  |  |
| *Lyciasalamandra billae* | H23 | **MN854825** | **MN867152** | **MN867275** |  |  |
| *Lyciasalamandra billae* | H24 | **MN854826** | **MN867153** | **MN867276** |  |  |
| *Lyciasalamandra billae* | H25 | **MN854827** | **MN867154** | **MN867277** |  |  |
| *Lyciasalamandra billae* | H26 | **MN854828** | **MN867155** | **MN867278** |  |  |
| *Lyciasalamandra billae* | H27 | **MN854829** | **MN867156** | **MN867279** |  |  |
| *Lyciasalamandra billae* | H3 | **MN854809** | **MN867136** | **MN867259** |  |  |
| *Lyciasalamandra billae* | H4 | **MN854810** | **MN867137** | **MN867260** |  |  |
| *Lyciasalamandra billae* | H5 | **MN854811** | **MN867138** | **MN867261** |  |  |
| *Lyciasalamandra billae* | H6 | **MN854812** | **MN867139** | **MN867262** |  |  |
| *Lyciasalamandra billae* | H7 |  |  |  | **EU430967** | **EU430929** |
| *Lyciasalamandra billae* | H8 |  |  |  | **EU430968** | **EU430930** |
| *Lyciasalamandra billae* | H9 |  |  |  | **EU430969** | **EU430931** |
| *Lyciasalamandra atifi* | H1 | **MN854718** | **MN867045** | **MN867168** |  |  |
| *Lyciasalamandra atifi* | H10 | **MN854726** | **MN867053** | **MN867176** |  |  |
| *Lyciasalamandra atifi* | H11 |  |  |  | **EU430959** | **EU430917** |
| *Lyciasalamandra atifi* | H12 | **MN854727** | **MN867054** | **MN867177** |  |  |
| *Lyciasalamandra atifi* | H12 |  |  |  | **EU430960** | **EU430918** |
| *Lyciasalamandra atifi* | H13 |  |  |  | **EU430961** | **EU430919** |
| *Lyciasalamandra atifi* | H14 |  |  |  | **EU430962** | **EU430920** |
| *Lyciasalamandra atifi* | H15 |  |  |  | **EU430963** | **EU430922** |
| *Lyciasalamandra atifi* | H16 |  |  |  | **EU430963** | **EU430923** |
| *Lyciasalamandra atifi* | H17 | **MN854728** | **MN867055** | **MN867178** |  |  |
| *Lyciasalamandra atifi* | H18 | **MN854729** | **MN867056** | **MN867179** |  |  |
| *Lyciasalamandra atifi* | H19 | **MN854730** | **MN867057** | **MN867180** |  |  |
| *Lyciasalamandra atifi* | H2 | **MN854719** | **MN867046** | **MN867169** |  |  |
| *Lyciasalamandra atifi* | H20 | **MN854731** | **MN867058** | **MN867181** |  |  |
| *Lyciasalamandra atifi* | H21 | **MN854732** | **MN867059** | **MN867182** |  |  |
| *Lyciasalamandra atifi* | H22 | **MN854733** | **MN867060** | **MN867183** |  |  |
| *Lyciasalamandra atifi* | H3 | **MN854720** | **MN867047** | **MN867170** |  |  |
| *Lyciasalamandra atifi* | H4 | **MN854721** | **MN867048** | **MN867171** |  |  |
| *Lyciasalamandra atifi* | H5 | **MN854722** | **MN867049** | **MN867172** |  |  |
| *Lyciasalamandra atifi* | H6 | **MN854723** | **MN867050** | **MN867173** |  |  |
| *Lyciasalamandra atifi* | H7 | **MN854724** | **MN867051** | **MN867174** |  |  |
| *Lyciasalamandra atifi* | H7 |  |  |  | **EU430962** | **EU430921** |
| *Lyciasalamandra atifi* | H8 | **MN854725** | **MN867052** | **MN867175** |  |  |
| *Lyciasalamandra fazilae* | H1 | **MN854734** | **MN867061** | **MN867184** |  |  |
| *Lyciasalamandra fazilae* | H10 | **MN854743** | **MN867070** | **MN867193** |  |  |
| *Lyciasalamandra fazilae* | H11 | **MN854744** | **MN867071** | **MN867194** |  |  |
| *Lyciasalamandra fazilae* | H12 | **MN854745** | **MN867072** | **MN867195** |  |  |
| *Lyciasalamandra fazilae* | H13 |  |  |  | **EU430972** | **EU430935** |
| *Lyciasalamandra fazilae* | H14 |  |  |  | **EU430973** | **EU430935** |
| *Lyciasalamandra fazilae* | H15 |  |  |  | **EU430972** | **EU430936** |
| *Lyciasalamandra fazilae* | H16 |  |  |  | **EU430974** | **EU430937** |
| *Lyciasalamandra fazilae* | H17 |  |  |  | **EU430975** | **EU430938** |
| *Lyciasalamandra fazilae* | H18 |  |  |  | **EU430976** | **EU430939** |
| *Lyciasalamandra fazilae* | H19 |  |  |  | **EU430976** | **EU430941** |
| *Lyciasalamandra fazilae* | H2 | **MN854735** | **MN867062** | **MN867185** |  |  |
| *Lyciasalamandra fazilae* | H20 | **MN854746** | **MN867073** | **MN867196** |  |  |
| *Lyciasalamandra fazilae* | H21 | **MN854747** | **MN867074** | **MN867197** |  |  |
| *Lyciasalamandra fazilae* | H22 | **MN854748** | **MN867075** | **MN867198** |  |  |
| *Lyciasalamandra fazilae* | H23 | **MN854749** | **MN867076** | **MN867199** |  |  |
| *Lyciasalamandra fazilae* | H24 | **MN854750** | **MN867077** | **MN867200** |  |  |
| *Lyciasalamandra fazilae* | H25 | **MN854751** | **MN867078** | **MN867201** |  |  |
| *Lyciasalamandra fazilae* | H26 | **MN854752** | **MN867079** | **MN867202** |  |  |
| *Lyciasalamandra fazilae* | H27 | **MN854753** | **MN867080** | **MN867203** |  |  |
| *Lyciasalamandra fazilae* | H29 | **MN854754** | **MN867081** | **MN867204** |  |  |
| *Lyciasalamandra fazilae* | H3 | **MN854736** | **MN867063** | **MN867186** |  |  |
| *Lyciasalamandra fazilae* | H4 | **MN854737** | **MN867064** | **MN867187** |  |  |
| *Lyciasalamandra fazilae* | H5 | **MN854738** | **MN867065** | **MN867188** |  |  |
| *Lyciasalamandra fazilae* | H5 |  |  |  | **EU430971** | **EU430934** |
| *Lyciasalamandra fazilae* | H6 | **MN854739** | **MN867066** | **MN867189** |  |  |
| *Lyciasalamandra fazilae* | H7 | **MN854740** | **MN867067** | **MN867190** |  |  |
| *Lyciasalamandra fazilae* | H8 |  |  |  | **EU430976** | **EU430940** |
| *Lyciasalamandra fazilae* | H8 | **MN854741** | **MN867068** | **MN867191** |  |  |
| *Lyciasalamandra fazilae* | H9 | **MN854742** | **MN867069** | **MN867192** |  |  |
| *Lyciasalamandra flavimembris* | H1 | **MN854755** | **MN867082** | **MN867205** |  |  |
| *Lyciasalamandra flavimembris* | H10 | **MN854761** | **MN867088** | **MN867211** |  |  |
| *Lyciasalamandra flavimembris* | H2 |  |  |  | **EU430979** | **EU430944** |
| *Lyciasalamandra flavimembris* | H3 |  |  |  | **EU430979** | **EU430945** |
| *Lyciasalamandra flavimembris* | H4 |  |  |  | **EU430979** | **EU430946** |
| *Lyciasalamandra flavimembris* | H4 | **MN854756** | **MN867083** | **MN867206** |  |  |
| *Lyciasalamandra flavimembris* | H5 |  |  |  | **EU430979** | **EU430947** |
| *Lyciasalamandra flavimembris* | H6 | **MN854757** | **MN867084** | **MN867207** |  |  |
| *Lyciasalamandra flavimembris* | H7 | **MN854758** | **MN867085** | **MN867208** |  |  |
| *Lyciasalamandra flavimembris* | H8 | **MN854759** | **MN867086** | **MN867209** |  |  |
| *Lyciasalamandra flavimembris* | H9 | **MN854760** | **MN867087** | **MN867210** |  |  |
| *Lyciasalamandra helverseni* | H1 | **MN854762** | **MN867089** | **MN867212** |  |  |
| *Lyciasalamandra helverseni* | H1 |  |  |  | **EU430970** | **EU430933** |
| *Lyciasalamandra helverseni* | H10 | **MN854771** | **MN867098** | **MN867221** |  |  |
| *Lyciasalamandra helverseni* | H11 | **MN854772** | **MN867099** | **MN867222** |  |  |
| *Lyciasalamandra helverseni* | H12 | **MN854773** | **MN867100** | **MN867223** |  |  |
| *Lyciasalamandra helverseni* | H13 | **MN854774** | **MN867101** | **MN867224** |  |  |
| *Lyciasalamandra helverseni* | H14 | **MN854775** | **MN867102** | **MN867225** |  |  |
| *Lyciasalamandra helverseni* | H15 | **MN854776** | **MN867103** | **MN867226** |  |  |
| *Lyciasalamandra helverseni* | H16 | **MN854777** | **MN867104** | **MN867227** |  |  |
| *Lyciasalamandra helverseni* | H17 | **MN854778** | **MN867105** | **MN867228** |  |  |
| *Lyciasalamandra helverseni* | H18 | **MN854779** | **MN867106** | **MN867229** |  |  |
| *Lyciasalamandra helverseni* | H19 | **MN854780** | **MN867107** | **MN867230** |  |  |
| *Lyciasalamandra helverseni* | H2 | **MN854763** | **MN867090** | **MN867213** |  |  |
| *Lyciasalamandra helverseni* | H20 | **MN854781** | **MN867108** | **MN867231** |  |  |
| *Lyciasalamandra helverseni* | H21 | **MN854782** | **MN867109** | **MN867232** |  |  |
| *Lyciasalamandra helverseni* | H3 | **MN854764** | **MN867091** | **MN867214** |  |  |
| *Lyciasalamandra helverseni* | H4 | **MN854765** | **MN867092** | **MN867215** |  |  |
| *Lyciasalamandra helverseni* | H5 | **MN854766** | **MN867093** | **MN867216** |  |  |
| *Lyciasalamandra helverseni* | H6 | **MN854767** | **MN867094** | **MN867217** |  |  |
| *Lyciasalamandra helverseni* | H7 | **MN854768** | **MN867095** | **MN867218** |  |  |
| *Lyciasalamandra helverseni* | H8 | **MN854769** | **MN867096** | **MN867219** |  |  |
| *Lyciasalamandra helverseni* | H9 | **MN854770** | **MN867097** | **MN867220** |  |  |
| *Lyciasalamandra luschani* | H1 | **MN854783** | **MN867110** | **MN867233** |  |  |
| *Lyciasalamandra luschani* | H1 |  |  |  | **EU430966** | **EU430928** |
| *Lyciasalamandra luschani* | H10 |  |  |  | **EU430965** | **EU430927** |
| *Lyciasalamandra luschani* | H11 | **MN854790** | **MN867117** | **MN867240** |  |  |
| *Lyciasalamandra luschani* | H12 | **MN854791** | **MN867118** | **MN867241** |  |  |
| *Lyciasalamandra luschani* | H13 | **MN854792** | **MN867119** | **MN867242** |  |  |
| *Lyciasalamandra luschani* | H14 | **MN854793** | **MN867120** | **MN867243** |  |  |
| *Lyciasalamandra luschani* | H15 |  |  |  | **EU430984** | **EU430942** |
| *Lyciasalamandra luschani* | H15 | **MN854794** | **MN867121** | **MN867244** |  |  |
| *Lyciasalamandra luschani* | H16 | **MN854795** | **MN867122** | **MN867245** |  |  |
| *Lyciasalamandra luschani* | H17 |  |  |  | **EU430984** | **EU430943** |
| *Lyciasalamandra luschani* | H18 |  |  |  | **EU430978** | **EU430942** |
| *Lyciasalamandra luschani* | H19 | **MN854796** | **MN867123** | **MN867246** |  |  |
| *Lyciasalamandra luschani* | H2 | **MN854784** | **MN867111** | **MN867234** |  |  |
| *Lyciasalamandra luschani* | H20 | **MN854797** | **MN867124** | **MN867247** |  |  |
| *Lyciasalamandra luschani* | H21 | **MN854798** | **MN867125** | **MN867248** |  |  |
| *Lyciasalamandra luschani* | H22 | **MN854799** | **MN867126** | **MN867249** |  |  |
| *Lyciasalamandra luschani* | H23 |  |  |  | **EU430980** | **EU430948** |
| *Lyciasalamandra luschani* | H24 |  |  |  | **EU430981** | **EU430949** |
| *Lyciasalamandra luschani* | H25 |  |  |  | **EU430982** | **EU430950** |
| *Lyciasalamandra luschani* | H26 |  |  |  | **EU430983** | **EU430951** |
| *Lyciasalamandra luschani* | H27 | **MN854800** | **MN867127** | **MN867250** |  |  |
| *Lyciasalamandra luschani* | H28 | **MN854801** | **MN867128** | **MN867251** |  |  |
| *Lyciasalamandra luschani* | H29 | **MN854802** | **MN867129** | **MN867252** |  |  |
| *Lyciasalamandra luschani* | H3 | **MN854785** | **MN867112** | **MN867235** |  |  |
| *Lyciasalamandra luschani* | H30 | **MN854803** | **MN867130** | **MN867253** |  |  |
| *Lyciasalamandra luschani* | H31 | **MN854804** | **MN867131** | **MN867254** |  |  |
| *Lyciasalamandra luschani* | H32 | **MN854805** | **MN867132** | **MN867255** |  |  |
| *Lyciasalamandra luschani* | H33 | **MN854806** | **MN867133** | **MN867255** |  |  |
| *Lyciasalamandra luschani* | H4 | **MN854786** | **MN867113** | **MN867236** |  |  |
| *Lyciasalamandra luschani* | H4 |  |  |  | **EU430983** | **EU430927** |
| *Lyciasalamandra luschani* | H5 | **MN854787** | **MN867114** | **MN867237** |  |  |
| *Lyciasalamandra luschani* | H6 | **MN854788** | **MN867115** | **MN867238** |  |  |
| *Lyciasalamandra luschani* | H6 |  |  |  | **EU430964** | **EU430925** |
| *Lyciasalamandra luschani* | H7 | **MN854789** | **MN867116** | **MN867239** |  |  |
| *Lyciasalamandra luschani* | H8 |  |  |  | **EU430964** | **EU430924** |
| *Lyciasalamandra luschani* | H9 |  |  |  | **EU430964** | **EU430926** |
