# Supplementary material for: Phylogeographic analyses point to long-term survival on the spot in micro-endemic Lycian salamanders
Source: PLoS One. 2020 Jan 13;15(1):e0226326. doi: 10.1371/journal.pone.0226326 (PMC6957296; doi:10.1371/journal.pone.0226326)
Supplement: S2 Table — (DOCX) [file pone.0226326.s008.docx]

**S2 Tab.** Results of Partition Finder with partitions and substitution models (cp = codon position).

| no. of partition | partition | substitution model |
| --- | --- | --- |
| 1 | 2^nd^ cp ATP8 and 16S | HKY+I+G |
| 2 | 1^st^ cp ATP6 and 1^st^ cp ATP8 | TIM+G |
| 3 | 2^nd^ cp ATP6 | TrN+I+G |
| 4 | 3^rd^ cp ATP6 and 3^rd^ cp ATP8 | TrN+G |
